# The breakthrough in protein structure prediction

**DOI:** 10.1042/BCJ20200963

**Published:** 2021-05-24

**Authors:** Andrei N. Lupas, Joana Pereira, Vikram Alva, Felipe Merino, Murray Coles, Marcus D. Hartmann

**Affiliations:** Department of Protein Evolution, Max Planck Institute for Developmental Biology, 72076 Tübingen, Germany

**Keywords:** AlphaFold2, artificial intelligence, CASP, deep learning, protein folding problem, protein structure prediction

## Abstract

Proteins are the essential agents of all living systems. Even though they are synthesized as linear chains of amino acids, they must assume specific three-dimensional structures in order to manifest their biological activity. These structures are fully specified in their amino acid sequences — and therefore in the nucleotide sequences of their genes. However, the relationship between sequence and structure, known as the protein folding problem, has remained elusive for half a century, despite sustained efforts. To measure progress on this problem, a series of doubly blind, biennial experiments called CASP (critical assessment of structure prediction) were established in 1994. We were part of the assessment team for the most recent CASP experiment, CASP14, where we witnessed an astonishing breakthrough by DeepMind, the leading artificial intelligence laboratory of Alphabet Inc. The models filed by DeepMind's structure prediction team using the program AlphaFold2 were often essentially indistinguishable from experimental structures, leading to a consensus in the community that the structure prediction problem for single protein chains has been solved. Here, we will review the path to CASP14, outline the method employed by AlphaFold2 to the extent revealed, and discuss the implications of this breakthrough for the life sciences.

One of the key pieces of information required to understand a biological process is the structure of its constitutive proteins, but experimental approaches to structure determination are often time-consuming, laborious, and have uncertain outcomes, requiring large investments of time and resources. In contrast, protein sequences are readily obtained by translating genomic sequence and are available in great abundance. Since the structure of a protein is fully specified by its sequence, attempts to deduce one from the other — known as the protein folding problem — have been ongoing for half a century, rising in importance with the exponential growth in sequence databases and in frustration with the succession of methods that failed to bring decisive advance. Indeed, starting with the first decade of this century, there was a growing realization in the protein science community that this problem was one of the grand challenges of computational biology [1].

Things did not start this way. The secondary structures modeled by Linus Pauling from stereochemical considerations of the polypeptide chain [2,3] and soon afterwards the demonstration that such secondary structures could be assembled into three-dimensional models for α-keratin [4,5] and collagen [6,7] led to the expectation that a combination of geometric considerations, model-building, and parametric equations could solve the principles of protein structure, as they had already done for nucleic acids. However, the first protein crystal structures and their astonishing irregularity gave way to the realization that these principles might be considerably more complex than expected [8].

Despite this, excitement at the beginning of the 1990s about progress achievable through simplified biophysical representations of the polypeptide chain [9,10] and threading [11,12] led to the perception of rapid, often decisive, advance in deducing structure from amino acid sequence. This was, however, not matched by real-life applications of these methods, and it became apparent that some of the reported successes might have been due to ‘postdiction’, that is to the prediction of targets whose structure was already known to the predictors. To obtain an objective assessment of the state of the art in protein structure prediction, a group of scientists led by John Moult of the University of Maryland organized an experiment in 1994, CASP (for critical assessment of structure prediction), in which predictors could evaluate their methods within a doubly blind framework [13]. The organizers would collect sequences of proteins whose structure was not released (and in some cases had not even been fully determined yet) and make them available as prediction targets to computational scientists. The organizers would then hand the submitted predictions and solved structures to assessors, who had no knowledge of the participating prediction teams behind the group numbers. At the end of the experiment, which was to be repeated every two years, there would be a conference at which the results would be discussed.

CASP1 was a sobering experience, as the tools for structure prediction turned out to be quite blunt. In the words of the organizers: ‘Plenty went wrong with these predictions, and therein lies the principal value of the experiment’ [13]. The one source of information that worked reliably was the structure of proteins related to the target, and targets having relatives of known structure were classified as the easiest, accessible through modeling on the homologous template (called template-based modeling or TBM). However, it turned out to be quite hard to make a model that was closer to the target than the nearest available template because of errors in detection and target-to-template alignment. Applying biophysical methods, such as energy minimization, only seemed to make errors worse. Correspondingly, CASP2 saw an increased investment in the detection, modeling, and refinement of more remote homologs [14,15]. CASP2 was more successful than CASP1, particularly in the harder targets lacking detectable templates in the structure database (called free modeling or FM) where CASP1 predictions had been essentially random, but progress was still limited. The New York Times famously headlined its report with ‘Proteins 1, Computer 0’ and quoted one of the organizers, who had seen small signs of progress, that ‘it's encouraging but still a very long way from anything that could be useful’, while one of the assessors quipped that ‘failure can no longer be guaranteed’ [16].

From this base, the CASP3-5 experiments offered further improvements, however, mainly in the area of targets with intermediate difficulty, where a succession of increasingly powerful sequence search tools [17–19] allowed for the detection of ever more distant homologs. As it turned out, homologous proteins retain substantially the same fold, even when their sequences have seemingly diverged into a ‘midnight zone’ of dissimilarity [20]. In contrast, efforts to incorporate biophysical parameters into the prediction methods [21], while providing some impressive successes for smaller targets, did not scale to larger ones, leaving the statistical detection of evolutionary relatedness the main tool of structure prediction.

A measure for model accuracy introduced in CASP3, GDT-TS (for global distance test-total score), allowed to compare outcomes within and between experiments [22]. After iterative superposition of two structures with identical sequence, this measure compares the positions of cognate Cα carbons, tallies the percentage of pairs falling within distance cutoffs of 1, 2, 4, and 8 Å, adds up the percentages and divides them by four. This focus on similarity allows the measure to distinguish models that are poor, but contain locally correct segments, from globally wrong models, something that is not achieved by other related measures, such as root-mean-square deviation. Very roughly, GDT-TS scores around 20 denote largely random models, scores around 50 models with overall correct topology, and scores around 70 models with accurate global and local topology. Above 80, details of the structure are increasingly modeled correctly and above 95, models are as accurate as models built from experimental data.

An overview of GDT-TS scores in individual CASP experiments, shown as polynomial fits through the best scores achieved for each target in that experiment, was presented by John Moult in the introduction to the CASP14 conference, slide 19 [23] (the slide is also found at https://en.wikipedia.org/wiki/AlphaFold#/media/File:CASP_results_2020.png). This overview shows that, after CASP5, overall progress largely came to a standstill until CASP12, leading some in the field to wonder whether we would ever get to a solution of the problem. The seeds of the next advance, however, had already been sown.

From the start of the CASP experiments, scientists had wondered whether it would be possible to compute inter-residue contact maps from correlated mutations in the multiple sequence alignments of homologous proteins, in order to obtain a fingerprint of the fold and guide structure prediction [24]. The idea was that if mutations were correlated, the residues at those positions were most likely in physical contact, providing the sort of information that allows structure determination by NMR. The accuracy of such contact maps remained low for many years, however, because analyzing correlations in a pairwise manner could not distinguish between direct, structural correlations and indirect, functional ones. Much better distinction between the different sets of co-evolving residues became possible from around 2010 onward by global contact predictions using direct coupling analysis [25–27], which considered all pairwise interactions simultaneously and optimized the contact map globally to the observed set of pairwise correlations. This approach took a further, major step forward in 2017 with the demonstration that deep learning methods could not only extract high-quality contact maps from multiple alignments in this way, even in cases with few homologs, but also interpret the predicted contacts into a set of distances that allowed for a finer-grained geometric fingerprint for the underlying fold [28]. The application of convolutional neural networks for distance map prediction was used by leading structure prediction groups in CASP13 (2018) and had a powerful effect on the hard targets, for which the GDT-TS of the best models went from around 40 to over 60 (see slide 19 in [23]).

Among the groups scoring highly in CASP13 was an unexpected newcomer, AlphaFold, fielded by DeepMind, the leading artificial intelligence laboratory of Alphabet Inc. To everybody's surprise, this group bested all participants with the key insight that the probability distribution of the distance map could be converted to a protein-specific statistical potential, which could generate the protein fold by minimization [29,30]. While AlphaFold's lead in CASP13 was larger than the typical distance between the first- and second-ranked groups at previous CASP experiments, its overall performance was more incremental than transformational, providing the best model in only about a third of the cases, albeit with a larger lead for harder targets than for easier ones (Figure 1).

**Figure 1.**
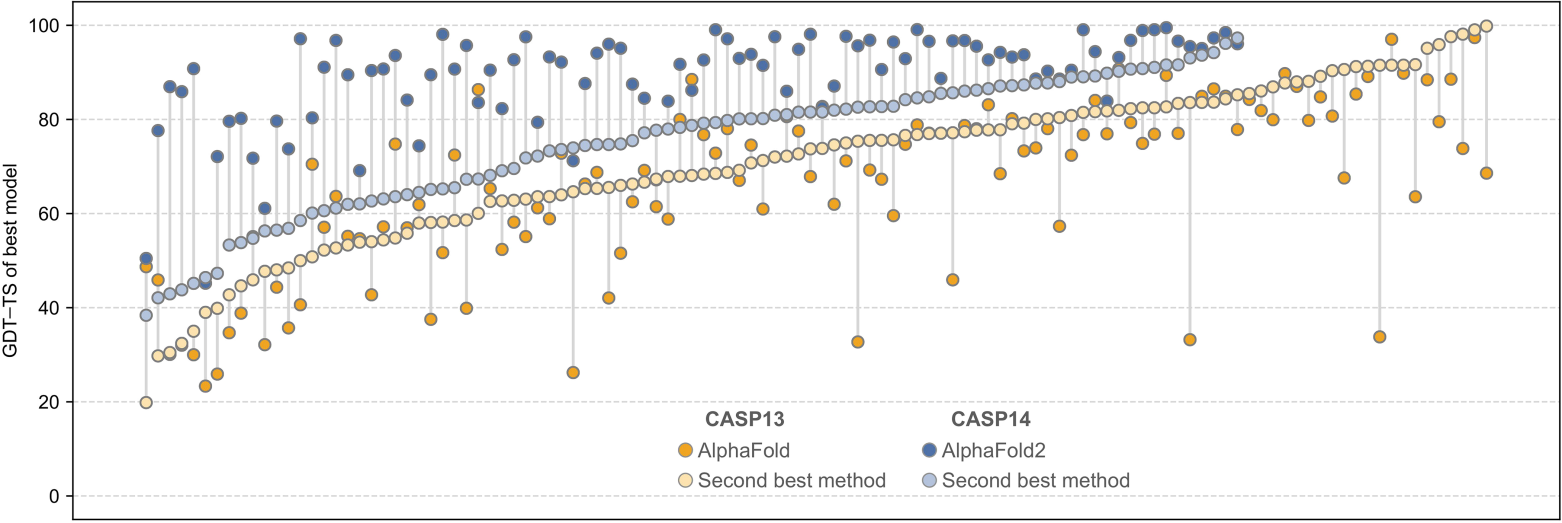
Graph illustrating the predictive success of AlphaFold in CASP13 (orange; darker dots; 114 models) and AlphaFold2 in CASP14 (blue; darker dots; 93 models) relative to the best models entered by any other group (lighter dots). The latter are ordered along the *x*-axis by ascending GDT-TS. The graph was inspired by the blog of Mohammed AlQuraishi [29].

No one was, therefore, prepared for the transformational performance of AlphaFold's second incarnation, AlphaFold2, at CASP14, where it placed far ahead of all other participants, achieving a median GDT-TS of 92.4 for its predictions! To recall, this is in the range of experimental structures, leading many to conclude that the structure prediction problem for single protein chains was now solved, as stated by John Moult in his concluding remarks to the CASP14 conference. A comparison of AlphaFold2 predictions with the best models submitted by other groups (Figure 1) makes the extent of the advance clear, as AlphaFold2 predictions usually had GDT-TS scores >80 even for the hardest targets (structure correct in most details), while the second-best models for these targets were below 60 (overall topology correct).

As an illustration of this, we would like to briefly recount the case of target T1100, an archaeal transmembrane receptor, for which AlphaFold2 submitted a model with GDT-TS around 80 and the next best groups models with GDT-TS around 55 (Figure 2). Our group entered this target as a result of an online meeting of organizers and assessors in August 2020, at which the astonishing predictions of group 427 (revealed later to be AlphaFold2) were brought to a point succinctly by Nick Grishin, one of the assessors: ‘So, either this group is close to solving the folding problem or they cheated somehow’. In response, we mentioned that we had diffraction data for a transmembrane receptor, which we had failed to solve for almost a decade because of phasing problems. Would group 427 file models sufficiently good to solve the dataset by molecular replacement? Surely there was no way to cheat on this. The short of it is that the structure could be solved readily with the AlphaFold2 models. Other submitted models had good overall topology, but many local departures from the structure, making them poor templates for molecular replacement (Figure 2). As an interesting side aspect of this, 12 of the 20 highest-ranking groups for this target submitted the co-ordinates of a public prediction server as their best answer, occasionally with minor attempts at refinement. The server, tFold, is run by the AI laboratory of the Chinese technology company Tencent, showing that DeepMind is not the only company laboratory interested to enter the fray.

**Figure 2.**
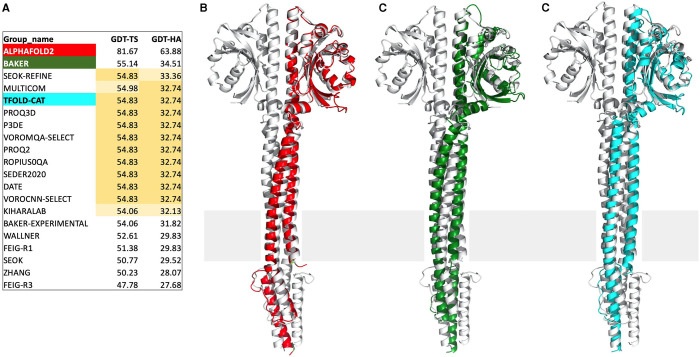
Target T1100, the transmembrane receptor Af1503 from the archaeon *Archaeoglobus fulgidus,* with the expected location of the membrane indicated by a gray bar. (**A**) The top 20 groups for this target are ranked by GDT-HA (the high-accuracy version of GDT that penalizes larger deviations from the reference structure by only considering pairs of cognate Cα carbons at distance cutoffs of 0.5, 1, 2, and 4 Å, half the cutoffs used by GDT-TS). AlphaFold2 (red), whose model was used to solve the experimental dataset by molecular replacement, is clearly leading the field by a wide margin, ahead of the best human group (Baker; green) and the best server (tFold; cyan). The co-ordinates predicted by the tFold server, which is public, were filed by 11 other groups as the best prediction (highlighted in ochre), occasionally with minor tweaks (light ochre). (**B**) Superposition of the AlphaFold2 model (red), (**C**) of the Baker model (green), and (**D**) of the tFold model (cyan) onto the crystal structure (gray).

What allowed AlphaFold2 to build this commanding lead? A more detailed evaluation will have to await the publication of the method in the CASP14 proceedings, but from the presentation of John Jumper for the AlphaFold2 team at the CASP14 conference [31] and the opinion of experts in the field [29,32], the architecture of the prediction network has changed in fundamental ways. Whereas AlphaFold used convolutional neural networks for distance map prediction and applied gradient descent optimization to construct models from these restraints, AlphaFold2 built an end-to-end network for which the model parameters could be tuned jointly, from the sequence input to the structure output, in order to optimize the final model instead of proxy measures along the way. Such end-to-end training for network optimization was proposed by Mohammed AlQuraishi after CASP13 [33] and was shown here to be an important component in predictive success. Furthermore, AlphaFold2 used attention modules to derive distance constraints and built structural models from them with 3D equivariant transformer neural networks, which operate directly on atoms in three-dimensional space. Attention modules, which originated in natural language processing (for an excellent presentation on them see [34]), do not derive summary statistics from the input multiple sequence alignment, but choose a subset of sequences to focus on and derive a first distance map, on the basis of which they decide which sequences to focus on in the next iteration. In this way, through iterative optimization (which may have required more than a hundred rounds in some cases), the network can extract a richer set of constraints even from sequence alignments that contain few full-length homologs, accounting for its particularly impressive performance on hard targets relative to all other methods. The overall strategy of this network architecture seems to aim for the best local solutions in order to assemble the global model from them and this has clearly been highly successful.

So, has DeepMind solved the protein folding problem? In its basic form — deducing the native structure of a protein from its amino acid sequence — the answer from CASP14 appears to be yes for most proteins, provided the program has access to the protein sequence and structure databases, and the target protein is folded. Objections that a solution implies understanding or that the prediction is not made from the single amino acid sequence boil down to semantics, in our opinion. However, the protein folding problem is more complex than just deducing a static three-dimensional structure from a sequence. A protein sequence not only contains the information for the structure, but also for the path by which this structure will be reached, for the dynamic adjustments it will undergo in response to changing conditions and binding partners, for the components of the cellular machinery it will need to engage to reach its native location. From the information in its sequence, a protein can recognize its binding partners (copies of itself, other proteins, cellular structures such as the membrane, small molecules) and know whether it will alter these by catalysis or through conformational changes, and whether it will fold or unfold conditionally upon encountering them. All these aspects, which are currently outside the scope of AlphaFold2, are essential for the biological function of proteins and scientists are understandably most excited about them. We would therefore conclude that no, AlphaFold2 was not the last step towards solving the protein folding problem, but rather the first step on a very exciting new path towards goals in protein structure prediction that may now have come within reach.

Does this mean that the advance obtained by AlphaFold2 has been hyped and is in fact not all that impressive? Definitely no to this as well. We find that the advance is absolutely astounding, something we have stressed repeatedly in our contributions to the CASP14 media coverage (see for example [35,36]). We think that the long, arduous journey to this breakthrough, involving some of the brightest minds in biophysics and computational biology, is ample evidence for the magnitude of this achievement. Indeed, the need to introduce deep learning methods for this advance prompts us to ask whether the structure prediction problem may have been too hard for the human mind to solve. Paraphrasing J.B.S. Haldane, who suspected that the universe is not only stranger than we suppose, but stranger than we *can* suppose, might the problem have been harder than we *could* have solved?

We fear that this is the case and that one of the reasons for the success of end-to-end training is the elimination of human bias. Decades of efforts by highly trained scientists and many billions of dollars in public investment clearly produced the data needed to breach the problem, but the breakthrough required computational networks which, unlike the human brain, were optimized for the analysis of non-linear correlations. Like so many other groups — athletes and chess players to name two — we will have to become used to the fact that machines have capabilities beyond our biological range. We look forward to what we think will be a wave of advanced prediction servers, both from the leading academic groups and from companies with advanced machine learning capabilities, which will make the structure space of proteins as broadly and rapidly accessible as BLAST did for the sequence space 25 years ago, marking a similar revolution in the life sciences.

## Open Access Statement

Open access for this article was enabled by the participation of Max Planck Institute for Developmental Biology in an all-inclusive *Read and Publish* pilot with Portland Press and the Biochemical Society under a transformative agreement with Max Planck Digital Library.

## Abbreviation

CASP: critical assessment of structure prediction
GDT-TS: global distance test-total score
